# Supplementary material for: Comparative Analysis of Deep Learning Models for Predicting Causative Regulatory Variants
Source: bioRxiv. 2025 Jun 11:2025.05.19.654920. Preprint. [Version 2] doi: 10.1101/2025.05.19.654920 (PMC12190767; doi:10.1101/2025.05.19.654920)
Supplement: Supplement 1 [file media-1.pdf]

Comparative Analysis of Deep Learning Models for Predicting Causative Regulatory Variants – Supporting Information

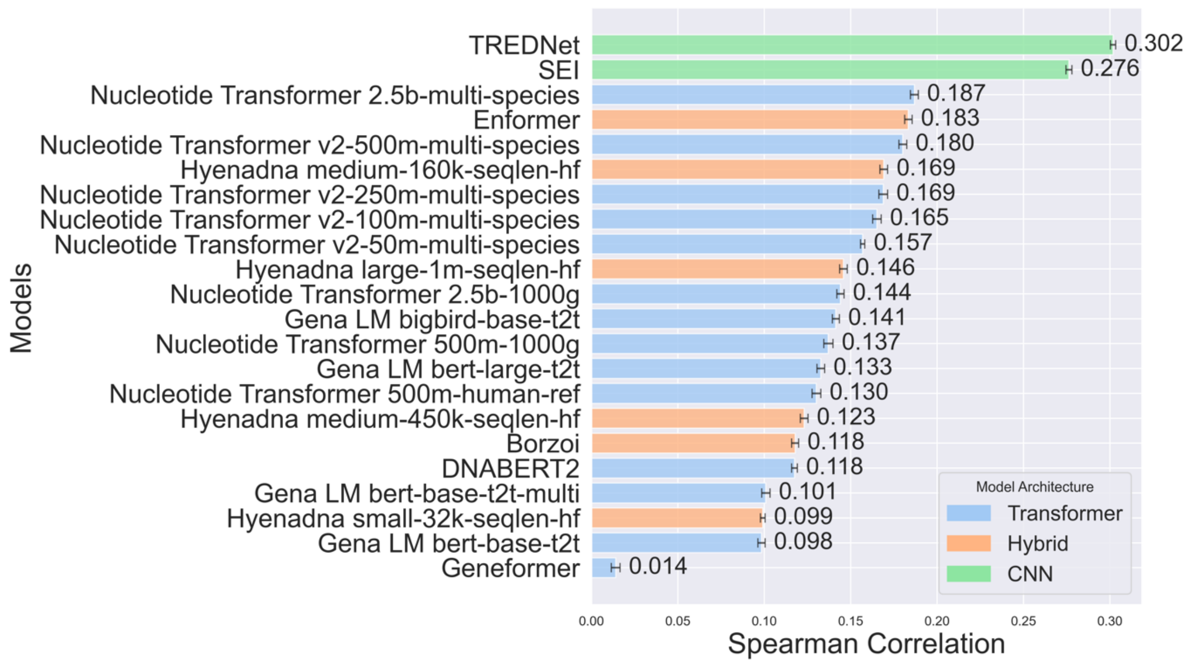

Figure S1. *Spearman correlation between model predictions and experi-mental log2-fold changes for enhancer variant effects across the human genome. Bar colors denote model architectures (CNN: green, transformer: blue, and hybrid: orange). All correlations have p-values < 0.05, and error bars show variance.*

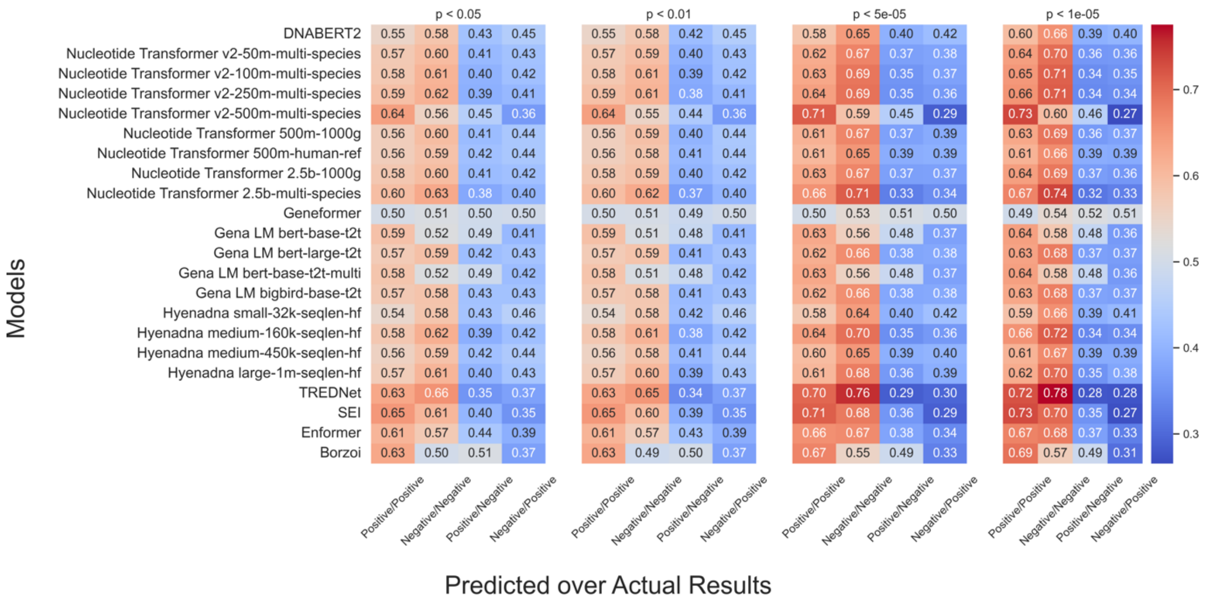

Figure S2. *Heatmap of model variant predictions (Predicted) versus experimental values (Actual Results) at different p-value thresholds, highlighting performance variations in identifying positive/negative outcomes across architectures.. The color intensity represents the fraction of values predicted as positive/negative relative to the experimental positive/negative values. Red indicates higher fractions (desired in the first two columns from the left), while blue indicates lower fractions (desired in the last two columns).*

Table S1. *Spearman correlation for various deep learning models across four cell lines: K562 (19321 SNPs), HepG2 (16255 SNPs), NPC (14042 SNPs), and HeLa (5241 SNPs). Bold and underline styles denote the top and second-highest correlations per cell line, respectively.*

	Cell Lines			
**Models**	**k562 (19321 SNPs)**	**hepg2 (16255 SNPs)**	**NPC (14042 SNPs)**	**Hela (5241 SNPs)**
DNABERT2	0.136255	0.13844	0.00983	0.10131
Nucleotide Transformer v2-50m-multi-species	0.193606	0.176066	0.029745	0.091993
Nucleotide Transformer v2-100m-multi-species	0.194085	0.210449	0.029202	0.085566
Nucleotide Transformer v2-250m-multi-species	0.230211	0.178415	0.031789	0.091625
Nucleotide Transformer v2-500m-multi-species	0.255502	0.193536	0.039783	0.076763
Nucleotide Transformer 500m-1000g	0.152783	0.193222	0.010707	0.06466
Nucleotide Transformer 500m-human-ref	0.158163	0.17474	0.016139	0.058858
Nucleotide Transformer 2.5b-1000g	0.167759	0.181192	0.014801	0.081246
Nucleotide Transformer 2.5b-multi-species	0.226941	0.22058	0.016179	0.091921
Geneformer	0.00589	0.030644	-0.00983	-0.009303
Gena LM bert-base-t2t	0.122552	0.116882	0.025676	0.052523
Gena LM bert-large-t2t	0.169897	0.142243	0.041452	0.084151
Gena LM bert-base-t2t-multi	0.096464	0.140658	0.015713	0.047429
Gena LM bigbird-base-t2t	0.169682	0.159723	0.023648	0.088436
Hyenadna small-32k-seqlen-hf	0.110639	0.129993	0.000771	0.075399
Hyenadna medium-160k-seqlen-hf	0.1927	0.208351	0.007788	0.118122
Hyenadna medium-450k-seqlen-hf	0.115686	0.186035	0.007832	0.093602
Hyenadna large-1m-seqlen-hf	0.162212	0.194539	0.012185	0.077734
TREDNet	**0.313784**	**0.365124**	**0.068044**	0.103394
SEI	<u>0.287206</u>	<u>0.315009</u>	<u>0.067235</u>	**0.155081**
Enformer	0.131957	0.230396	-0.005137	<u>0.129005</u>
Borzoi	0.089077	0.173757	0.024613	-0.019793

Table S2. *Pearson correlation coefficients for various deep learning models across multiple datasets (Data 1-9 and the respective SNPs). Bold and underline styles denote the top and second-highest correlations per cell line, respectively.*

**Model**	**Dataset 1** (19237 SNPs) K562	**Dataset 2** (14183 SNPs) HepG2	**Dataset 3** (1789 SNPs) HepG2	**Dataset 4** (84 SNPs) G562	**Dataset 5** (283 SNPs) HepG2	**Dataset 6** (14042 SNPs) NPC	**Dataset 7** (1692 SNPs) Hela	**Dataset 8** (1614 SNPs) Hela	**Dataset 9** (1665 SNPs) Hela
DNABERT2	0.086	0.098	0.049	0.140	0.137	-0.004	0.613	0.480	0.211
Nucleotide Transformer v2-50m-multi-species	0.147	0.104	0.129	0.178	0.322	0.021	0.575	0.354	0.218
Nucleotide Transformer v2-100m-multi-species	0.152	0.128	0.113	0.207	**0.570**	0.022	0.526	0.232	0.136
Nucleotide Transformer v2-250m-multi-species	0.166	0.111	0.217	0.142	0.431	0.042	-0.383	0.306	**0.329**
Nucleotide Transformer v2-500m-multi-species	0.199	0.116	-0.030	0.389	0.028	0.084	0.261	0.264	-0.010
Nucleotide Transformer 500m-1000g	0.123	0.119	0.111	0.325	0.068	0.000	0.532	0.346	0.162
Nucleotide Transformer 500m-human-ref	0.149	0.120	0.052	0.296	0.427	0.002	0.449	0.253	0.218
Nucleotide Transformer 2.5b-1000g	0.147	0.113	0.165	0.238	0.182	0.004	0.607	0.357	0.188
Nucleotide Transformer 2.5b-multi-species	0.153	0.140	0.340	0.311	0.491	0.060	0.557	0.366	0.066
Geneformer	0.005	0.028	0.029	0.228	0.121	0.004	-0.108	-0.021	0.031
Gena LM bert-base-t2t	0.077	0.050	-0.008	0.151	-0.138	0.047	-0.465	0.216	0.031
Gena LM bert-large-t2t	0.117	0.089	0.192	0.203	0.234	0.041	0.754	0.099	0.017
Gena LM bert-base-t2t-multi	0.076	0.063	-0.058	0.156	-0.081	0.004	-0.400	0.057	0.107
Gena LM bigbird-base-t2t	0.136	0.100	0.291	0.412	-0.013	0.039	0.624	0.338	0.017
Hyenadna small-32k-seqlen-hf	0.084	0.100	0.104	0.174	0.265	0.019	0.293	0.015	0.251
Hyenadna medium-160k-seqlen-hf	0.149	0.144	0.267	0.289	0.123	-0.016	0.317	0.137	0.318
Hyenadna medium-450k-seqlen-hf	0.077	0.137	0.073	0.209	0.214	-0.036	0.630	0.166	0.238
Hyenadna large-1m-seqlen-hf	0.118	0.138	0.126	0.243	0.204	-0.016	0.606	0.116	0.227
TREDNet	**0.316**	**0.342**	0.397	**0.601**	0.363	0.167	0.784	0.410	0.085
SEI	0.298	0.298	0.394	0.341	0.051	**0.190**	0.801	**0.595**	0.237
Enformer	0.058	0.137	**0.492**	0.410	0.434	0.037	**0.843**	0.542	0.142
Borzoi	0.033	0.125	0.012	0.306	0.121	0.050	0.231	-0.237	-0.120

Table S3. *Spearman correlation for various deep learning models across multiple datasets (Data 1-9 and the respective SNPs). Bold and underline styles denote the top and second-highest correlations per cell line, respectively.*

Model	Dataset 1 (19237 SNPs) K562	Dataset 2 (14183 SNPs) HepG2	Dataset 3 (1789 SNPs) HepG2	Dataset 4 (84 SNPs) G562	Dataset 5 (283 SNPs) HepG2	Dataset 6 (14042 SNPs) NPC	Dataset 7 (1692 SNPs) Hela	Dataset 8 (1614 SNPs) Hela	Dataset 9 (1665 SNPs) Hela
DNABERT2	0.136	0.136	0.103	0.168	0.105	0.018	0.627	0.513	0.336
Nucleotide Transformer v2-50m-multi-species	0.194	0.170	0.191	0.348	-0.018	0.029	0.273	0.496	0.252
Nucleotide Transformer v2-100m-multi-species	0.194	0.203	0.222	0.135	<u>0.382</u>	0.016	0.545	0.442	0.069
Nucleotide Transformer v2-250m-multi-species	0.231	0.171	0.306	0.196	0.372	0.027	0.309	0.507	0.186
Nucleotide Transformer v2-500m-multi-species	0.256	0.187	-0.020	0.442	0.226	0.079	0.264	0.432	-0.089
Nucleotide Transformer 500m-1000g	0.153	0.188	0.148	0.429	-0.077	0.014	0.345	0.390	0.064
Nucleotide Transformer 500m-human-ref	0.158	0.170	0.062	<u>0.528</u>	0.066	0.007	0.309	0.416	0.111
Nucleotide Transformer 2.5b-1000g	0.168	0.173	0.285	0.488	0.355	0.016	0.582	0.495	0.104
Nucleotide Transformer 2.5b-multi-species	0.228	0.209	0.342	0.400	0.305	0.040	0.427	0.517	0.072
Geneformer	0.006	0.030	0.044	0.330	-0.076	0.007	0.027	-0.039	-0.017
Gena LM bert-base-t2t	0.122	0.115	0.066	0.285	0.180	0.059	-0.518	0.394	0.091
Gena LM bert-large-t2t	0.170	0.135	0.221	0.510	0.360	0.047	0.555	0.448	0.199
Gena LM bert-base-t2t-multi	0.096	0.140	0.012	0.410	-0.138	0.019	-0.035	0.242	0.157
Gena LM bigbird-base-t2t	0.170	0.155	0.287	0.512	0.090	0.037	0.482	0.428	0.178
Hyenadna small-32k-seqlen-hf	0.111	0.126	0.200	0.391	0.146	0.011	0.545	-0.096	<u>0.371</u>
Hyenadna medium-160k-seqlen-hf	0.193	0.201	0.310	0.487	0.178	-0.009	0.409	0.461	**0.428**
Hyenadna medium-450k-seqlen-hf	0.116	0.181	0.147	0.274	0.167	-0.013	0.655	0.376	0.326
Hyenadna large-1m-seqlen-hf	0.162	0.188	0.182	0.453	0.270	-0.010	<u>0.700</u>	0.406	0.370
TREDNet	**0.314**	**0.360**	0.416	**0.573**	**0.435**	<u>0.135</u>	0.673	0.536	0.267
SEI	<u>0.287</u>	<u>0.309</u>	<u>0.427</u>	0.495	0.149	**0.148**	0.627	**0.633**	0.248
Enformer	0.132	0.218	**0.566**	0.374	0.267	-0.016	**0.900**	<u>0.553</u>	0.053
Borzoi	0.088	0.172	0.036	0.270	0.173	0.073	0.282	-0.368	0.009

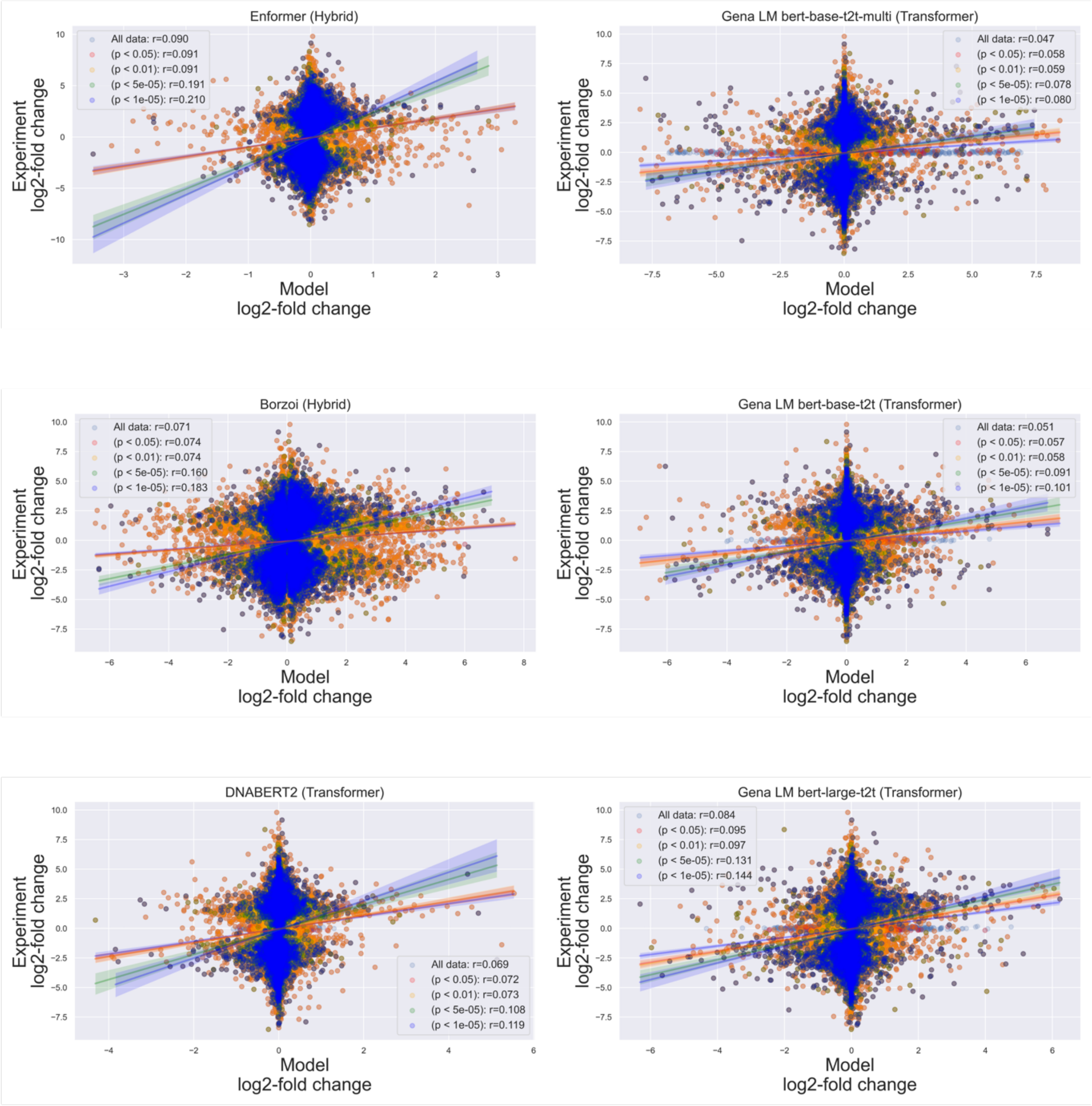

Figure S3a*: Model performance relative to experimental data significance, using top models from each architecture category.*

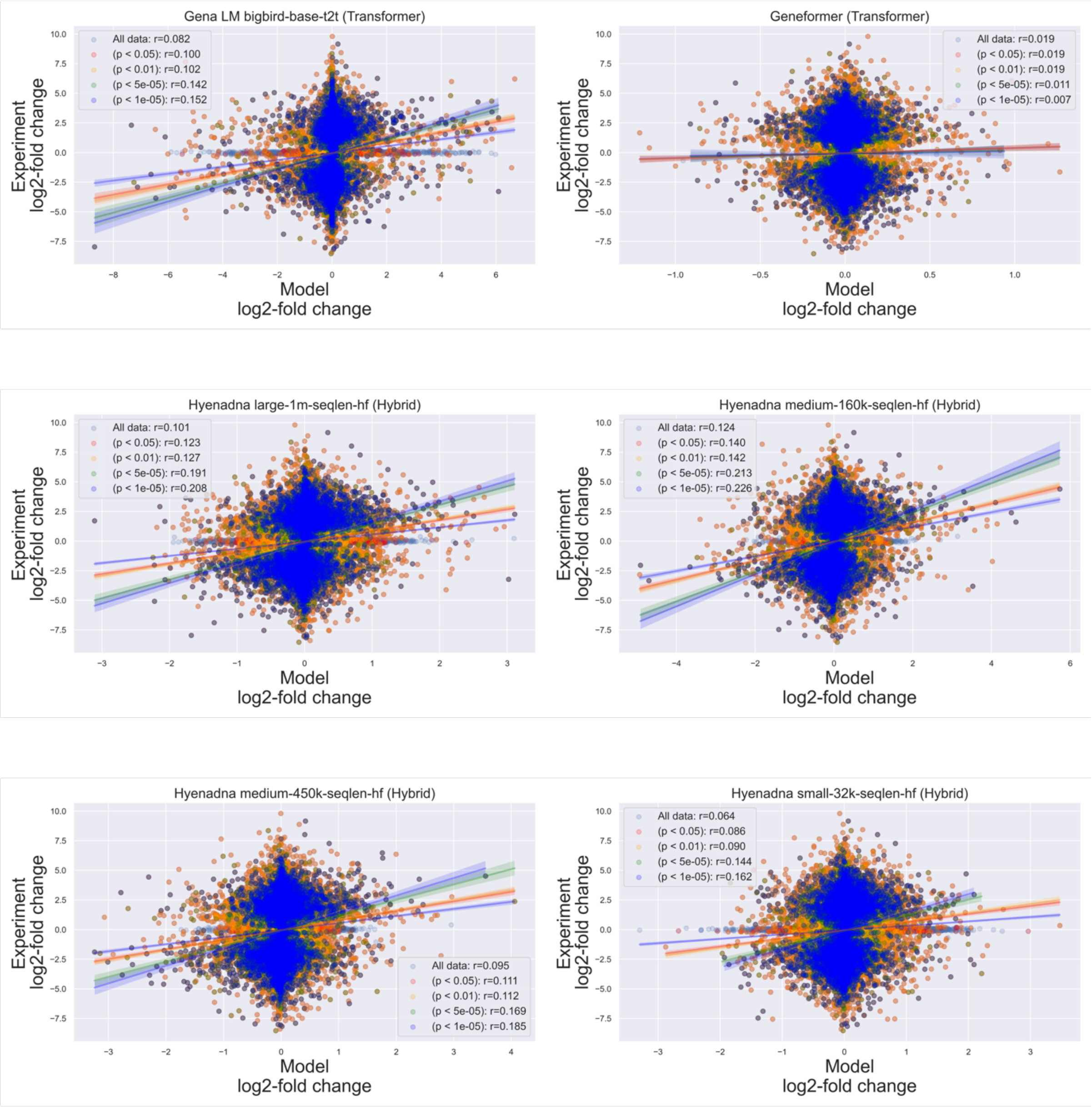

Figure S3b: *Model performance relative to experimental data significance, using top models from each architecture category.*

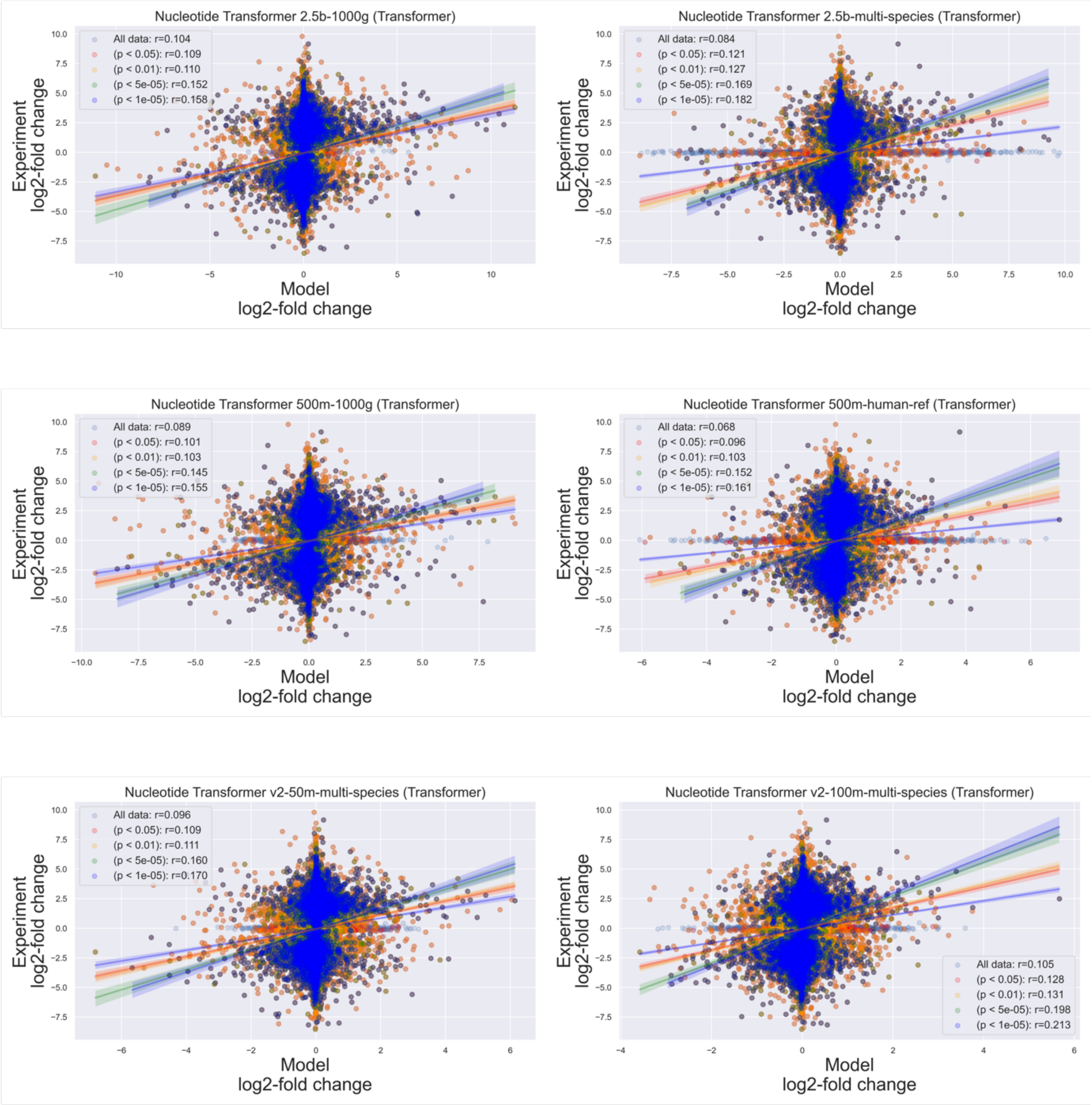

***Figure S3c**: Model performance relative to experimental data significance, using top models from each architecture category.*

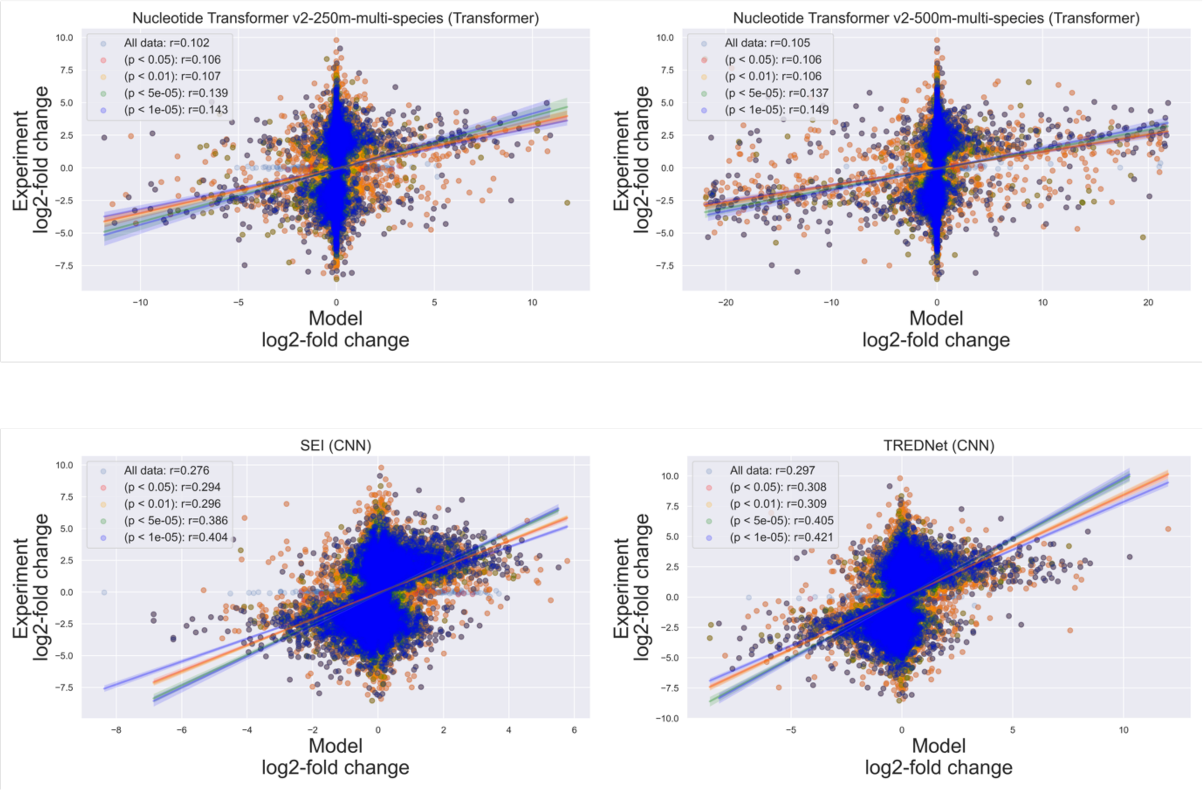

***Figure S3d**: Model performance relative to experimental data significance, using top models from each architecture category.*

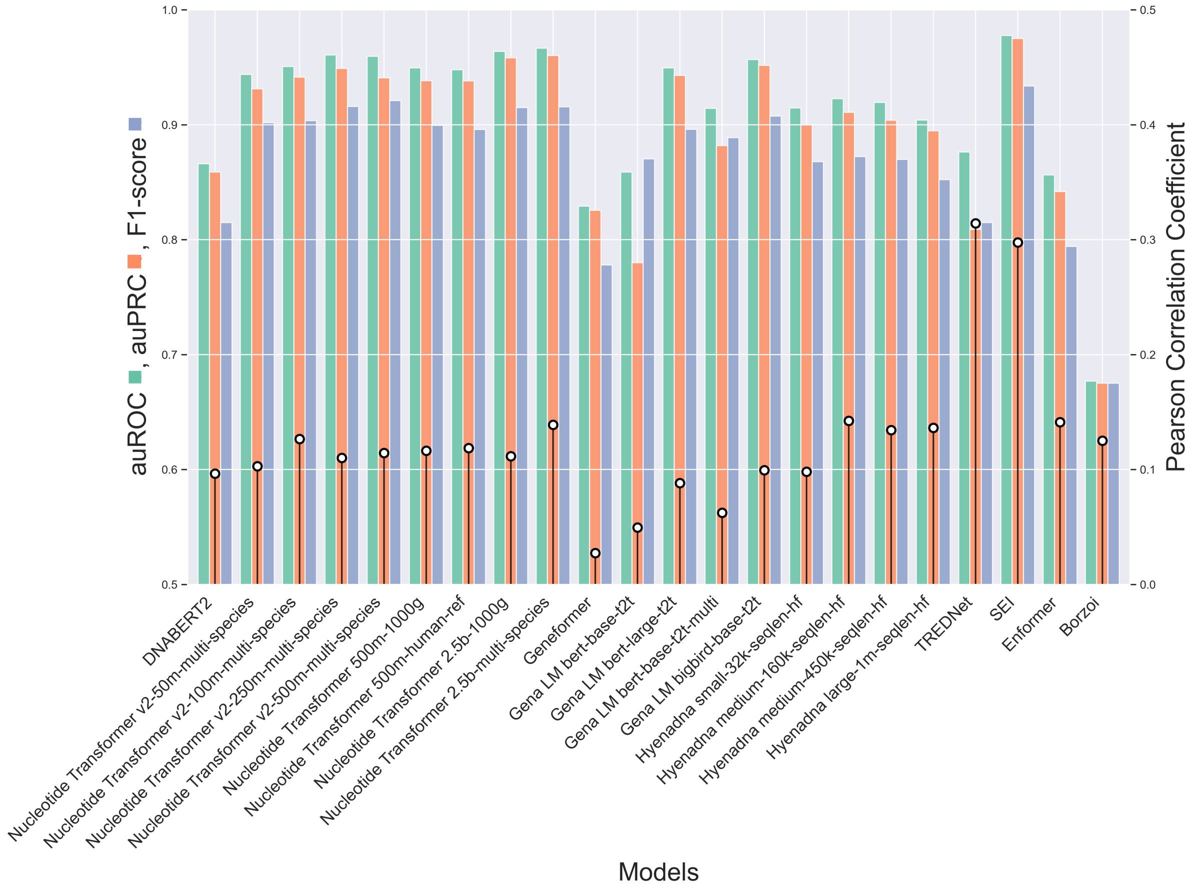

***Figure S4**: Performance comparison of different models for enhancer detection in HepG2 cell across multiple metrics. Left axis: Area Under the Receiver Operating Characteristic Curve (auROC, green bars), Area Under the Precision-Recall Curve (auPRC, orange bars), and F1-score (blue bars). Right axis: Pearson Correlation Coefficient (black pins).*
